# The impact of social networking services on the coronavirus disease 2019 (COVID-19) pandemic in sub-Saharan Africa

**DOI:** 10.11604/pamj.supp.2020.35.2.23073

**Published:** 2020-06-08

**Authors:** Francky Teddy Endomba, Jean Joel Bigna, Jean Jacques Noubiap

**Affiliations:** 1 Psychiatry Internship Program, University of Bourgogne, 21000 Dijon, France; 2 Health Economics & Policy Research and Evaluation for Development Results Group, Yaoundé, Cameroon; 3 Department of Epidemiology and Public Health, Centre Pasteur of Cameroon, Yaoundé, Cameroon; 4 Centre for Heart Rhythm Disorders, University of Adelaide and Royal Adelaide Hospital, Adelaide, Australia; 5 Ministry of Public Health, Yaoundé, Cameroon

**Keywords:** COVID-19, social networking services, sub-Saharan Africa

## Abstract

Social networking services played a crucial role in the management of previous outbreaks around the world. African populations are increasingly using social networks and this may have benefits but also harmful consequences, especially at this time of coronavirus disease 2019 pandemic. This paper concisely discusses of these consequences which include the propagation of “fake news” and the misinterpretation of messages pertaining to the prevention and the treatment of the disease. Moreover, our commentary provides some ways to alleviate them, chiefly represented by a framed and practical communication by health authorities. We suggest for instance the systematic sharing of correct messages through official Facebook and Twitter accounts and the conception of tailored web tools dedicated to the verification of circulating information.

## Commentary

Coronavirus disease 2019 (COVID-19) that emerged from China in December 2019 has become a pandemic and a global public health crisis [1]. Along with other means to curb the pandemic, social networking services (SNS) can have major impact. SNS are crucial educational and information canal that can help raising the population awareness about the disease and disseminate information about preventive measures and processes to seek care for suspected cases [2]. Importantly, SNS can maintain a permanent link between the population and government and health authorities, ensuring day-to-day updates on the global and local progression of the outbreak and control strategies [2,3]. Noteworthy, in the situation of confinement and quarantine, SNS help to preserve a social link between people and consequently, to prevent the negative psychological impact of social isolation [4]. However, besides these benefits, SNS might have serious drawbacks including the rapid propagation of fear and anxiety across the population,[4] as well as wrong information commonly known as “fake news” [5].

Africa is also affected by the pandemic, with a potentially important health, social and economic shock that is expected in the coming weeks or months [1]. Social media have a special role to play in the COVID-19 crisis in Africa. SNS are increasingly used on the continent. In 2019, 216 million people (16.6% of the total population) were actively using SNS in Africa, corresponding to a 13% increase from the previous year [6]. The mainly used social platforms by African populations are Facebook, WhatsApp and Facebook Messenger, and it should be noticed their emergent use of Twitter [6]. The COVID-19 seems to dominate the content of communication via SNS at this critical moment. On top of the abovementioned benefits, SNS is a particularly useful tool in the current pandemic in Africa [3]. For instance, in countries like Senegal or Rwanda, amongst several others, WhatsApp chatbots are used to provide reliable information and help in selecting people for rapid COVID-19 testing [7]. In Cameroon, through a twitter account, the minister of health provides timeously updates regarding the epidemiologic situation in the country, along with advice. In a similar manner, information pertaining to the COVID-19 pandemic evolution in Africa is daily presented by the concerned World Health Organization (WHO) Regional Office as by the different WHO national offices in Africa. Also, in addition to its preventive role regarding social isolation (same role as WhatsApp and Facebook Messenger), Facebook is widely used by the general public and health care workers to communicate around the disease, especially on preventive measures [2].

On the other hand, major concerns have been raised about SNS use in Africa in this period. In populations in which the literacy rate is low, fake news can spread very rapidly [5]. For instance, a huge number of posts on Facebook and WhatsApp have relayed statements that several natural products and traditional medicine are effective against COVID-19 [8]. Although the efficacy of such simple and widely available products in Africa would be absolutely beneficial for the fight against COVID-19, there is no evidence to support it at this point. Therefore, it might be disastrous if people in the community with symptoms of COVID-19 treat themselves with such traditional medications instead of seeking conventional medical care. This could be associated with delayed treatment, increased mortality and massive community transmission of the disease. Another critical issue is the misinterpretation of information regarding the use of chloroquine for the treatment of COVID-19 [5,9]. The disputed debate on the matter in France has spread to Francophone countries, and lot people see chloroquine as a miracle drug. As a result, many people have started self-medication with chloroquine for preventive and curative purposes, with many cases of overdose that have been reported so far [9]. More recently, a scandal was provoked by inappropriate comments on using vulnerable African populations for trials of BCG against COVID-19, made by two researchers on a popular television in France [10]. This has rapidly spread within populations in francophone countries via SNS. Although, this has been condemned by the WHO and several scientific authorities [10], unfortunately, it has tarnished the image of vaccination potentially in a significant number of people; with some health care workers reporting non-adherence to vaccination by many parents since then.

Only an intense and proactive communication by health authorities can effectively mitigate the effects of fake news via SNS during the COVID-19 crisis. To achieve it, populations should be educated about the benefits and risks of SNS in such a context. Furthermore, health authorities should intensify the sharing of correct information and educational materials through tailored communication pathways including Short Message Service (SMS), messages through SNS (i.e. on official Facebook and Twitter accounts), in official languages and local dialects. Likewise, the establishment of websites ensuring the verification of circulating information and adapted to sub-Saharan African populations, could be helpful to control “fake news” dissemination.

## Competing interests

The authors declare no competing interests.
